# A framework for capacity enhancement of Rwandan nurse educators and preceptors facilitating nursing students to learn pediatric pain management

**DOI:** 10.1186/s12912-024-01769-4

**Published:** 2024-02-17

**Authors:** Philomene Uwimana, Donatilla Mukamana, Yolanda Babenko-Mould, Oluyinka Adejumo

**Affiliations:** 1 School of Nursing and Midwifery, University of Rwanda, KG 11 Ave 47 St, 3682 Kigali, Rwanda; 2 Arthur Labatt Family School of Nursing, University of Western Ontario, 1151 Richmond St, N6A 3K7 London, ON Canada

**Keywords:** Capacity enhancement, Framework, Learning facilitation, Nurse educators, Pediatric pain management, Preceptors, Rwanda

## Abstract

**Introduction:**

In low- and middle- income countries like Rwanda, children are most likely to suffer from painful and life-limiting conditions due to various factors predominant in these settings. Adequate pre-licensure pain management training can improve pain relief nursing practices. Educators and nurses in practice have a responsibility to teach the soon-to- be nurses for holistic competent nursing care of children, emphasizing the importance of and addressing child’s suffering, yet inconsistencies were reported in what was taught regarding pediatric pain management from a theoretical perspective by nurse educators as compared to their counterparts in clinical settings in Rwanda.

**Design:**

This study applied a qualitative approach using group discussions based on nominal group technique (NGT) to develop and validate a conceptual framework supporting the capacity enhancement of nurse educators and preceptors facilitating students’ learning about pediatric pain management in Rwanda.

**Methods:**

NGT meetings were conducted with academic nurses (*n* = 8), nurse clinicians (*n* = 4), and nursing students (*n* = 2) during a 2-day workshop to identify strategies, discuss the relevance of each strategy and to agree on key concepts of a framework for improving the ability of nurse educators and preceptors to teach PPM to nursing students. After four weeks another round of NGT meetings were done with nursing faculty (*n* = 6), academic nurse leaders (*n* = 3), nurse preceptors (*n* = 2), clinical nurse leaders (*n* = 2), a nurse leader from a regulatory body (*n* = 1), and nursing student (*n* = 1) to validate the developed framework.

**Findings:**

Four main strategies corresponding to the key concepts were mapped in a framework. Participants had a consensual agreement on the importance of the developed framework, they confirmed its completeness and practicality. In addition, participants found that the developed framework is logical, and confirmed that it is applicable for its purpose.

**Conclusion:**

The developed framework presents an opportunity to respond to the gaps in nursing pain education in the context of limited resources settings such as Rwanda. It can also be applied in nursing practice and nursing research, aligning with the need of improving the quality of care of suffering children. Furthermore, the framework can be adapted and utilized to meet the needs of healthcare professionals other than nurses.

**Clinical relevance:**

Strategies are suggested to improve the ability of nurse educators and preceptors in clinical settings to facilitate the acquisition of pediatric pain management competencies by the next generation of nurses. Future Rwandan nurses could then use the competencies acquired to provide optimal health care to children with pain in the best way possible during their practices.

**Supplementary Information:**

The online version contains supplementary material available at 10.1186/s12912-024-01769-4.

## Introduction

Effective pain management remains a serious problem in over 150 countries where an estimated 80% of the global population including children, are affected by a lack of adequate pain management [1]. Among other barriers to proper pain management, the authors mentioned barriers related to the healthcare system, healthcare professionals, and to patients. They further indicated that from literature, healthcare professionals had below-average knowledge and skills for pain management [1]. In Rwanda, existing literature outlined inadequate pain management mainly due to limited access to pain medication, technologies and training for healthcare professionals [2, 3]. Also previous study reported the cultural influence as a factor affecting pain management, whereby people tend to hide their pain and children encouraged to be stoic, bearing with the suffering (“*kwihangana*”), which could impact on the decision to manage pain or not [4]. As part of a multidisciplinary team of healthcare professionals, nurses play a crucial role in effective pain management. Hence, nurses should be knowledgeable and pain-sensitive as they are involved in several aspects of pain management, including assessment and reassessment, planning and implementation of pain relief interventions, monitoring and reporting the impact of the interventions, and providing and reinforcing patients’ education [5, 6]. Pediatric pain management (PPM) by nursing staff can be ineffective due to a variety of issues that range from competency deficiency to structural issues in an organization, and cultural factors [7–10].

A review of the literature highlighted that adequate pain management education at the pre-service (pre-licensure) stage can improve pain relief practices [11–14]. However, the few studies conducted with nurse educators involved in facilitating learning about pain management in pre-service education revealed knowledge gaps among nurse educators [15, 16].

The National Council of Nurses and Midwives (NCNM) in Rwanda stipulated that nurse educators as well as nurses in practice have a responsibility to prepare students to holistically practice competent nursing care of children with an understanding of the importance of and how to alleviate a child’s suffering [17], yet nursing students have reported inconsistency in what they had been taught regarding PPM by nurse educators from a theoretical perspective as compared to preceptors in clinical settings. Also, nurse educators and preceptors have reported recognition of their knowledge gaps which were attributed to lack of or inadequate pain management education during their pre-service learning experiences [4]. Furthermore, teaching and learning about PPM was impacted by factors including lack of PPM information integrated into the overall curriculum or particular teaching plans; negligence of children’s pain in clinical settings; inadequate support to students; facilitators’ attributes; collaboration between academics and clinicians; nurses’ limited autonomy for decision-making regarding PPM practices, and shortage of human and material resources [4, 18]. Subsequently, in the current study, the authors sought to develop a suitable framework that could respond to the needs of nurse educators and preceptors for enhancing their capacity to facilitate students’ learning about PPM.

Therefore, the focus of this paper is to present a validated framework that was conceptualized and developed to support the capacity enhancement of nurse educators and preceptors facilitating students’ learning about PPM.

## Methods and materials

This study was part of a multi-phases mixed methods research study that adopted both pragmatic and post-positivism paradigms, and in which the steps of the intervention research design and development (IR:D&D) [19] were adapted. The IR:D&D was applied for identifying key concepts, designing, developing, and for the validation of the framework. This paper reports findings from a qualitative approach that used nominal group technique (NGT). NGT was applied in this study, seeking key strategies for capacity enhancement of nurse educators and preceptors facilitating nursing students’ competence acquisition for PPM. The researchers opted for NGT because it helps to obtain information from participants who have expert insight into a given topic and aims to reach a general agreement on that particular topic of interest [20]. The steps of NGT that were followed in this study included an introduction and presentation of the question, a silent generation of ideas, a ‘round-robin’ i.e., a brainstorming strategy on a specific topic, discussion in the plenum of a large group, and agreement about identified key concepts [21].

Two rounds of NGT were conducted, the first round consisted of a 2-day workshop of NGT meetings and was aimed to elicit key strategies for capacity enhancement of nurse educators and preceptors who facilitate competence acquisition by nursing students for PPM, whilst in the second round of NGT a 1-day workshop was conducted where the researchers sought to validate through a consensus of participants on the relevance, the context, and the structure of the framework developed to enhance the capacity of nurse educators and preceptors to facilitate nursing students’ learning about PPM.

Participants were recruited based on purposive and also convenience sampling that consisted of involving participants who were readily available and willing to participate, but also who could provide rich information due to their experience [22]. Participants included nurse academicians, nurse clinicians, nurse leaders, and nursing students. These categories of participants brought their contribution, each one within his/her position and expertise by providing accurate information meant to guide and support the achievement of the purpose of the study. The academicians were nurse educators with experience of over 5 years in teaching pediatric nursing courses either in the classroom, in skills-lab, and/or in clinical settings. Also, among academicians, there were academic leaders at school and department levels. The participants from clinical settings included matrons of pediatric units and pediatric nurses with extensive experience in caring for hospitalized children with pain and supporting students’ learning in clinical practice. The students were finalists in either advanced or bachelor of nursing programs who completed the course of pediatric nursing. Additionally, one person from the nursing regulatory council in Rwanda participated in this study. The research team was comprised of members with expertise in qualitative research methods, child health care, and nursing education. Table 1 illustrates the number and profile of participants in the first and second rounds of NGT.

**Table 1:** Characteristics of participants in the NGTs

Item	Participant characteristics	Number of participants	
		NGT round1 (*n* = 14)	NGT round2 (Validation workshop) (*n* = 15)
Category	Nurse educator	5	6
	Head of department	1	2
	Dean	2	1
	Nursing students	2	1
	Nurse preceptors	2	2
	Matron of pediatric unit (pediatric nurse specialist)	2	2
	Nurse Leader (Nursing and Midwifery Council)		1
Institution	Academic	10	10
	Clinical practice setting	4	4
	Nursing regulatory body		1
Qualification	PhD	2	1
	Master’s degree	9	11
	Bachelor degree	1	2
	Student in Advanced Diploma	1	
	Student in Bachelor of nursing	1	1
Participated in two rounds of NGT			9

In the first round, the NGT meetings followed the steps as described in [21] and were facilitated by one researcher (PU) who informed participants about the objective of the NGTs and the expected outcome and obtained their signed consent. PU presented an overview of the findings and conclusions emanating from the previous phase of the multi-phase study. Handouts were provided to participants as references for those who did not take notes during the presentation. The main tasks that guided participants during the process included: (1) *Identify issues considered as important in facilitating students’ competency acquisition for PPM*, and (2) *Determine strategies that will be appropriate to deal with the identified issues.* Other subsequent questions were (3) *to specify who can be involved, when, how, and where; (4) State the expected outcomes for the identified strategies*. Participants were requested to write their ideas on a notepad given to them for the purpose. This was the silent generation of ideas. In the small groups, ‘round-robin’ was conducted, and one group member captured on a flipchart all the group members’ ideas. They identified a list of issues that were pertinent to the facilitation of students’ learning about PPM. The same steps were repeated to identify strategies for dealing with the listed issues. In a plenary session of the large group, further discussion about each item presented was done to ensure a common understanding. Duplicate items were eliminated and each agreed-on item was listed on a flipchart. A consensus was obtained through a ranking from participants as they determined a list of what they considered to be key strategies for capacity enhancement of nurse educators and preceptors facilitating students’ learning about PPM in Rwanda. Participants also used colored post-it notes to show which key strategy was related to the other. It is from that list of key strategies that participants were asked to select and rank the top ones from which the core concepts were drawn to design a framework to enhance the capacity of nurse educators and preceptors facilitating nursing students learning PPM. This interactive analysis allowed to determine the outcome of the first round of NGT meetings as it provided a sense of accomplishment, capturing participants’ final decision regarding the initial questions.

A second workshop that was conducted four weeks after the first round of NGT was facilitated by DM and lasted 5 h. After being introduced to the objective of the workshop, participants reviewed a document presented by PU regarding the process and the outcome of the previous NGT. This was done in a session of about 2 h in which participants were requested to look at the logical flow of the process, the completeness of the information, and if the outcome was valid and reflected what came from the consensus of participants during the previous NGT meetings. In addition, participants looked at further steps performed by the researchers (PU, DM & OA) that led to the identification of the key concepts used to design the framework and establish the appropriateness of the identified key concepts. The second session of the workshop lasted 3 h where participants examined how the key concepts were mapped and discussed the structure of the diagram representing the framework. After discussions on identified modifications that could be done on the key concepts and the structure of the conceptual framework, an agreement was reached on the suggestions for further refinement. For the framework validation, the researchers developed a tool that consisted of a 5-point Likert scale with statements that included strongly disagree, disagree, agree, strongly agree, and a response area requesting participants to provide any comment or suggestion relating to the designed framework. Participants used the tool to evaluate the framework based on a set of criteria such as the importance, logical development, completeness and clarity, logical structure, applicability, validity, and practicality of the framework, which criteria were adopted from the literature [23], and then data were analyzed content analysis.

## Findings

The findings indicated that from a consensual process of NGT, seven issues were identified as most pertinent to the facilitation of nursing students learning about PPM. These issues are inadequate curriculum; lack of preparedness for nurse educators and preceptors; inadequate resources; cultural misconception of pain and its management; lack of policy and guidelines about PPM; lack of decision-making autonomy for PPM among nurses; and poor collaboration between nurse educators and preceptors.

Considering the Rwandan context, suggestions about strategies to deal with the issues were deliberated among participants and each identified issue was aligned to a strategy. Agreed on strategies included: a review of the undergraduate nursing curriculum to integrate core competencies for PPM; improved knowledge and practice on PPM for nurse educators and preceptors; securing resources for PPM at teaching and clinical sites; correct pain misconceptions and promote positive attitudes regarding PPM; provision of PPM policy and guidelines; empowering nurses for clinical decision-making toward PPM and improving collaboration between teaching institutions and clinical sites. Furthermore, participants indicated key players to be involved in the implementation of the strategies, at which institutional level and the expected outcomes. Details concerning the issues impacting the facilitation of students’ learning PPM as well as the key strategies for handling those issues as identified by the consensus of participants in the first round of NGTs are depicted in supplement Table 1. Further synthesis was done in the following session of NGT that led to mingle strategies that could respond to more than one issue, and the researchers looked at concepts that best capture statements of strategies leading to the same output. In this regard, the proposed strategy related to increasing knowledge and practice of nurse educators and preceptors on PPM was combined with the one of correcting pain misconceptions and promoting positive attitudes regarding PPM to enhance patient safety culture into PPM competency development. Also, the provision of a PPM policy and securing adequate resources for PPM concur with empowering nurses for clinical decision-making toward PPM. Therefore, four categories of strategies were retained, corresponding to key concepts that include curriculum enhancement, competence development for PPM, nurses’ empowerment, and collaboration reinforcement between nurse educators and preceptors. Table 2 indicates the merging of strategies and the maintained key concepts.

**Table 2:** Identified key concepts

Strategies identified by consensus in NGTs	Key concepts
Review of the existing curriculum in undergraduate nursing programs	Curriculum enhancement
Increase knowledge, attitude, and practice of nurse educators and preceptors on PPM.	PPM competence development
Correct pain misconceptions and promote positive attitudes for PPM (patient safety culture)	
Develop, review, and disseminate PPM policy, guidelines, and protocols (PPM in quality care improvement indicators)	Nurse empowerment
Empowering nurses for clinical decision-making toward PPM	
Securing resources for PPM	
Improving collaboration between nurse educators and preceptors	Collaboration reinforcement

### Contextual definition of key concepts

#### Curriculum enhancement

This refers to reviewing the nursing curriculum and enriching it with all the essential information related to pediatric pain and its management to be taught and/or learned, and the required means for teaching and learning PPM. Also, curriculum enhancement is meant to integrate expected competencies for PPM to be achieved after the nursing program, making it more comprehensive and holistic. Hence, academicians are expected to review and enhance the curricula that provide appropriate guidance to educators to elaborate suitable teaching materials i.e., content to teaching and choosing adapted teaching/learning approaches in the appropriate learning time for the expected PPM competencies.

#### PPM competence development

This concept refers to the required interventions for improving the knowledge, attitudes, and practices of nurse educators and preceptors towards PPM. This should start from the pre-licensing training where graduating students are equipped with competencies for PPM before entering professional practice, and be continued as in-service training to update them on the evolving evidence practices for PPM. Also, PPM competence development implies senior nurses mentoring newly graduated ones and formative supervision from a direct manager, and sensitization for correcting misconceptions on pediatric pain while promoting positive attitudes toward PPM. Therefore, managers at academic institutions and in clinical settings, and nurse educators and preceptors themselves should invest in these essential aspects for the development of PPM competence.

#### Nurse empowerment

Nurse empowerment is conceptualized as providing nurse educators and preceptors with the power and authority of making decisions related to children’s pain relief and implementing those decisions within their scope of practice. In this context, empowerment covers the aspects relating to the availability, dissemination, and implementation of policy, guidelines, and protocols for PPM; to the promotion of interprofessional collaboration in the care of suffering children; and the availability of adequate resources for training at the teaching institution and for assessing and managing pediatric pain in the clinical area.

### Collaboration reinforcement

This refers to improving the cooperation and partnership between nurse educators and preceptors for the continuity of students’ facilitation to acquire competence for PPM. Also, it refers to sharing knowledge and lived experiences related to current best practices for PPM among nurse educators and preceptors and implies consistency in PPM competencies taught to nursing students by nurse educators and preceptors. This can be easily done through a close collaboration between academic institutions and health facilities.

With these key concepts, a framework was designed for the capacity enhancement of nurse educators and preceptors to facilitate nursing students’ learning PPM.

#### Conceptual framework

##### The context

The framework was developed referring to the nursing perspective in Rwanda. The framework provides a foundation to support capacity enhancement to nurse educators and preceptors to facilitate nursing students’ competency acquisition for pediatric pain management. The framework can be used by academic and practice-based administrators, policy-makers, nurse educators, and preceptors for the aforementioned purpose. It can also be used by senior nurses in clinical settings to mentor and support newly graduated nurses. The framework is flexible and can be applied for the capacity development of other healthcare professionals.

### The structure of the framework

**Fig. 1:**
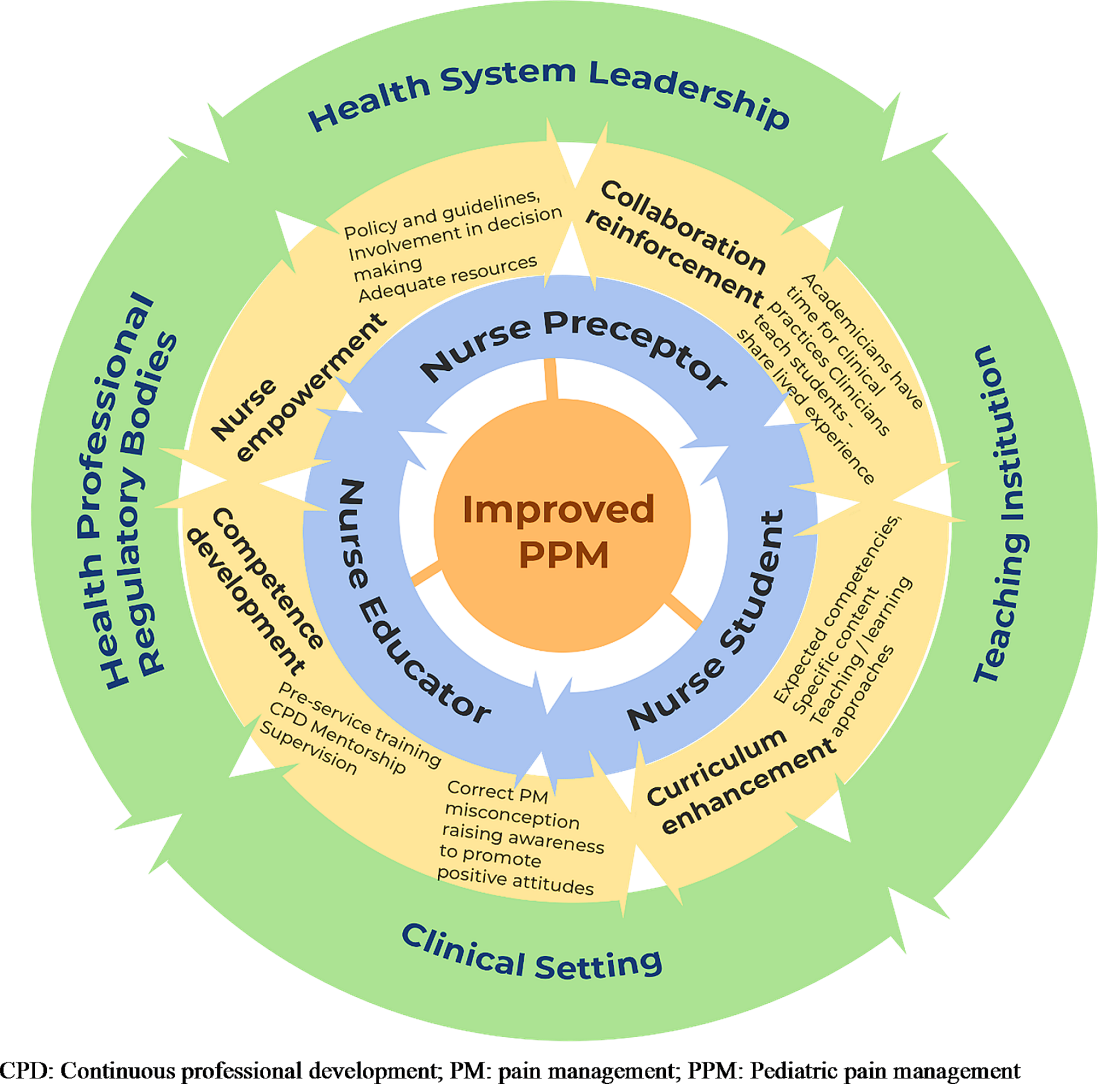
Dynamic nursing pediatric pain management competence facilitation framework

The framework is represented by a diagram in a form of a wheel shape with bi-directional arrows indicating a dynamic connection between all the players involved in the process of PPM competency acquisition by nursing students and the key strategies that are linked to one another (see Fig. 1). The diagram has three layers showing the level of involvement and the required key strategies for enhancing the capacity of educators and preceptors.

The top layer indicates that teaching institutions and clinical settings together with health care system leadership and health professional regulatory bodies are all concerned with the development of nurse educators and preceptors toward the facilitation of PPM competence acquisition. Support from the leaders and managers of these institutions through the provision of appropriate resources and guidance plays an important role in strengthening the capacity of educators and preceptors to develop their ability and facilitate students to acquire competence for PPM.

The second layer of the diagram incorporates four major categories of concepts with functional elements under each key concept. Those are the key strategies and related interventions to enhance the capacity of nurse educators and preceptors to facilitate nursing students learning PPM. Also, these key strategies are interrelated and one key strategy informs the other one i.e., PPM competency development is achieved when the curriculum specifically indicates which competency is required, nurses need to be empowered and guided with policy to review a curriculum. Collaboration is needed in the review and implementation of the curriculum but also to sustain PPM competency development.

The third layer represents the interaction between the key stakeholders (nurse educators, preceptors, and students) who are directly concerned with the capacity to facilitate students’ learning. All the layers are dynamic and the whole process converges to the core outcome which is the improved PPM represented by the inner circle at the center of the diagram. Figure 1 depicts the graphic representation of the developed framework.

### Validation of the framework

During the validation workshop, most of the participants agreed that there was a logical flow in the process of developing the framework. All participants indicated that the key concepts which they found relevant as key strategies would allow educators and nurses in general, to facilitate competence acquisition for PPM. It also reflected the consensus in the previous workshop, indicating that the conceptual framework is relevant to be used for the intended purpose.

Recommendations were made to refine the structure and the visual presentation of the framework for more clarity. Starting by the level of involvement of decision-makers, participants recommended separating the health professional regulatory bodies and the health care system leadership and to keep it general for the sake of adaptability of the proposed framework. It was agreed that the framework could be adapted to other contexts different from Rwanda and by other health care professionals. At the level of the implementers, the participants recommended keeping the focus on nurses as the framework was developed specifically for their capacity enhancement for facilitating PPM competence acquisition by nursing students. Also to be consistent about nurse educator, nurse preceptor, and nurse student.

After agreeing on the adjustments, an evaluation tool was utilized to establish the suitability and practicality of the proposed framework. The feedback from participants revealed that the developed framework was regarded as feasible to implement whenever it is presented to policy-makers and applicable for enhancing the capacity of educators and preceptors and promoting effective facilitation of PPM competency acquisition by nursing students. Table 3 summarizes the feedback from participants in the validation workshop.

**Table 3:** Framework validation - Feedback from participants (*n* = 15)

Evaluation item	Strongly disagree	Disagree	Agree	Strongly agree	Comments
The framework is important	0 (0%)	0 (0%)	0 (0%)	15 (100%)	
The development of the framework is logical	0 (0%)	0 (0%)	2 (13.3%)	13 (86.7%)	
Clear definition of concepts and framework easy to understand	0 (0%)	0 (0%)	0 (0%)	15 (100%)	
Completeness	0 (0%)	0 (0%)	0 (0%)	15 (100%)	
The framework is structured in logical manner	0 (0%)	0 (0%)	4 (26.6%)	11 (73.4%)	to refine the structure and the visual presentation of the framework for more clarity
The framework is applicable for its purpose	0 (0%)	0 (0%)	0 (0%)	15 (100%)	very feasible whenever it is presented to policy makers
Validity	0 (0%)	0 (0%)	2 (13.3%)	13 (86.7%)	
Practicality	0 (0%)	0 (0%)	0 (0%)	15 (100%)	very practical as it is dynamic: integration of teaching in clinical and collaboration of stakeholders

## Discussion

This study developed a framework for supporting the capacity enhancement of nurse educators and preceptors to enable nursing students learning PPM competence. Participants identified key concepts fundamental for designing the framework including curriculum enhancement, competence development, nurse empowerment, and collaboration reinforcement.

A review of the nursing curricula was deemed crucial to integrate the required competencies for holistic PPM that nursing students nearing graduation should possess before entering the profession. As such, a comprehensive curriculum will guide the educators to elaborate appropriate teaching materials i.e., content to teaching and choosing adapted teaching/learning strategies in the appropriate learning time for the expected PPM competencies. Whereas curriculum was described as a social fact which is dynamic [24], teaching and learning strategies ought be adapted to the learning outcomes, hence it is required to update and enhance the curriculum to promote inspirational learning. Therefore, reviewing the nursing curricula is believed to strengthen foundational pre-service education on PPM in the Rwandan context by correcting the existing shortcomings in the content and teaching and learning approaches. This is in accord with [25, 26] claiming that pain management core competencies should be integrated into pre-licensure curricula to ensure that all nurses have the necessary knowledge and skills to help reduce pain as a public health crisis, and everyone in clinical practice should own pain education.

Strategies to increase knowledge and practice of PPM were organized around the concept of competence development. These strategies encompass pre-service education, in-service training, access to updated information on PPM, mentorship, supportive supervision from line managers, and regular clinical exposure for nurse educators. Apart from basic nursing education, engaging in post-licensure pain management education is regarded as crucial to building and maintaining competence [27]. Continuous education provides updates on best clinical practices to build on interaction skills and learning strategies for facilitating students’ optimal learning. Findings from the present study reiterated the need to address the issue of pediatric pain misconceptions and poor pain management among nurses through educational interventions aimed at promoting positive attitudes toward PPM.

Participants voiced that for effective PPM education and improvement of children’s pain relief practices, nurses in academia and clinical nurses should be empowered. Power was defined by Kanter [28] as the “ability to mobilize resources to get things done”, she asserted that the mandate of management is to create conditions for work effectiveness by ensuring employees have access to the information support, and resources necessary to accomplish effective work and that they are provided ongoing opportunities to develop [28]. As a result, there is an increase in employees’ levels of commitment to the organization, feelings of autonomy, and self-efficacy [29]. In this study, the concept of nurse empowerment encompasses the availability of clear guidance in terms of policies and protocols, the involvement of nurses in decision-making related to pain control interventions, and the availability of required resources. Local policies and protocols for PPM were recognized as essential for improving patients’ outcomes while maintaining the consistency of care and supporting healthcare professionals to make quality clinical decisions if they are well elaborated and validated [30]. From study participants’ perspectives, a policy on PPM and updated guidelines are utmost needed to refer to, for nurse educators to align with what they are teaching to future nurses and for clinical nurses to implement what is meant to be guiding a clinical decision for an effective PPM. Hence, formulation of PPM policy and reviewing the existing national pain management guidelines to include specific aspects of PPM is deemed pertinent.

Additionally, a refinement of the nursing scope of practice was believed crucial to determine nurses’ roles and responsibilities in PPM according to their qualifications. Our findings recommended involving nurses in collaborative practice for decision-making regarding pain relief practices instead of just implementing doctor’ orders. This was supported by previous literature [31, 32] highlighting the need to re-examine the organizational structure that promotes the culture of doctors-led care to empower nurses for clinical decision-making for PPM and make them interdependent with other professionals. Evidence showed when nurses are empowered, they can provide optimal care to patients, which makes their colleagues proud and may promote a sense of community. A particular attention should also be given to increasing the opportunities for interprofessional teaching and learning of healthcare professionals involved in the care of children. Securing adequate resources was mentioned as another contributing element to enhance the capacity of nurse educators and preceptors through the access and utilization of pain assessment and pain management resources. It is implicit that nursing students will have more chances to be facilitated in their learning process on PPM in settings where resources are available. This is congruent with previous studies [33] indicating that the availability of required resources could provide a conducive environment for optimal PPM.

The study findings indicated that reinforcement of collaboration between academic institutions and health facilities was important to facilitate knowledge sharing and collaborative practice among educators and clinical nurses, necessary to optimize students teaching and learning PPM. Similarly, Gordon, Watt-watson, & Hogans [34] indicated that the synergy between academicians and clinicians influences the outcomes of students’ learning activities. A positive and cooperative relationship with clinical nurses was beneficial for clinical teaching and for creating a good working environment for the clinical instructor who is unfamiliar with the unit [34]. Kaplow [35] stated that nurse competencies in the synergy model can be applied to the competencies of nurse educators. The author stressed the most important competencies that include clinical judgment, clinical inquiry, facilitation of learning, collaboration, caring practice, and advocacy, and moral agency [35]. Nurse educators and preceptors in Rwanda should nurture the mentoring relationship for the development of their competence and shared understanding of PPM. This will optimize the facilitation of students’ learning PPM while ensuring a continuum of effective pain management for suffering hospitalized children.

It is important to note that the implementation of the proposed framework for enhancing the capacity of nurse educators and clinical nurses to facilitate competence acquisition for PPM by nursing students requires active and synergetic involvement of key decision-makers. Hence, health care system leadership and health professional regulatory bodies are expected to provide clear guidance of what a competent professional nurse is entitled to for effective PPM. This includes policies, guidelines, a clear scope of nursing practices, and the availability of resources for PPM. Academic institutions and clinical facilities will refer to the policies and guidelines on PPM to review curricula and initiate interventions for nurses’ competence development. This aligns with those who stated that strategic leadership, networking, and partnership to share expertise and best practices are critical to improving nursing education in Sub-Saharan Africa [36]. It was further stated that it is essential to re-examine how to increase the capacity of educators and mentors, responsiveness of curricula, strong regulatory frameworks, and availability of infrastructure and resources for better quality, quantity, and relevance of nursing education [36], and this is equally relevant for improving PPM education in nursing specifically.

### Strengths and limitations of the study

The proposed conceptual framework is designed to contribute to improved quality healthcare provision through enhanced PPM education. The framework implies the involvement and interactions of key stakeholders in improving the capacity of nurse educators and preceptors to facilitate nursing students’ learning PPM including health system leaders, health professionals’ regulatory bodies, academic institutions, hospitals’ leaders, nurses, and students themselves. The fact that this study used a different source of information to determine significant concepts for the design of the framework constitutes its strength. The study participants were from various settings in different provinces of the country, including district hospitals, referral and university teaching hospitals, schools of nursing, health professional regulatory bodies. This study is limited by the fact that, like other qualitative studies, these findings cannot be generalized because they only reflect strategies to enhance the capacity of nurse educators and preceptors considering the perceived needs expressed by the study participants. The authors of this study recommend that the proposed conceptual framework should be further tested and validated at a large scale.

## Conclusion

This study proposed strategies for increasing nurse educators’ and preceptors’ capability to support nursing students learning PPM. The strategies are the concepts that helped to design a framework for nursing PPM competence facilitation. These concepts were organized around curriculum enhancement, competence development, nurse empowerment, and collaboration reinforcement. This framework reflects a set of interrelated strategies and indicates the interactions among key players at different levels i.e., from policy and decision-makers to the implementers, and beneficiaries of capacity enhancement for facilitation of nursing PPM competence. The proposed conceptual framework presents an opportunity to respond to the gaps in nursing pain education in the context of limited resources settings such as Rwanda as evidenced in previous research [37]. Also, it can be utilized in nursing practice and nursing research, aligning with the need of improving the quality of care of the suffering children. Furthermore, the proposed conceptual framework can be adopted and adapted to enhance the capacity of nurses and other health professionals facilitating competence acquisition in similar contexts of the current study.

### Electronic supplementary material

Below is the link to the electronic supplementary material.

Supplementary Material 1

## Data Availability

The datasets used and analysed during the current study are available from the corresponding author on reasonable request.

## Abbreviations

PPM: Paediatric Pain Management
NGT: Nominal Group Technique
NCNM: National Council for Nurses and Midwives
