# Supplementary material for: A framework for capacity enhancement of Rwandan nurse educators and preceptors facilitating nursing students to learn pediatric pain management
Source: BMC Nurs. 2024 Feb 17;23:127. doi: 10.1186/s12912-024-01769-4 (PMC10874038; doi:10.1186/s12912-024-01769-4)
Supplement: Supplementary file 1 — Supplementary Material 1 [file 12912_2024_1769_MOESM1_ESM.docx]

Supplement_ Table 1. Issues pertinent to the facilitation of students’ learning and strategies to enhance the capacity of nurse educators & preceptors facilitating students’ acquisition of competency for PPM as identified by participants’ consensus.

| **Important issues relating to the facilitation of competence acquisition in nursing students** | **Strategies to deal with the issues / how** | **Who/where** | **Expected outcome** |
| --- | --- | --- | --- |
| Inadequate curriculum: Not competence-based; lacks comprehensive and holistic management of pediatric pain | Review of the existing curriculum to include specified competencies for PPM  Teaching strategies and needed resources | Nurse academicians  Teaching institutions | A comprehensive curriculum with expected competencies for holistic PPM, and appropriate teaching materials (content, teaching strategies) |
| Lack of preparedness:  Limited knowledge and attitudes of nurse educators & Preceptors | Increase knowledge and practice of nurses on PPM   - Pre-service training - In-service training - Access to updated information on ppm - Mentorship and supervision - Seminars on PPM for graduating students | Teaching institutions  Hospital management  NCNM/RNMU  Nurse leaders (supervision and follow up) | Knowledgeable and pain-sensitive NE & Preceptors  Clinical mastering of PPM |
| Inadequate resources:  Limited simulation materials  Lack of pain assessment material in the hospitals  Shortage of nursing staff | Availing adequate resources for PPM:  -teaching institutions-- materials such as simulation equipment (manikins, simulated patients with pain scenarios, pain scales, protocols & guidelines, checklists…)  - in clinical settings ---- Advocate for the increase of nursing staff,  Material for pain assessment and management (pain scales, protocols & guidelines, medications, toys for children,…) | Management of  -Teaching institutions  -Hospital | Access and utilization of pain assessment and pain management resources |
| Cultural misconception of pain and its management:  Pain given less attention   - Pain is not avoidable - Pain expressed by patients and not only by measures - Pain misconception - Poor information on pain - Pain is undermined, poor attitude | Promote positive attitudes for PPM  - Clinical exposure for nurse educators  - Include pediatric pain management into quality care improvement indicators  Appoint the personnel in charge of PPM in service  - Mentorship  - supervision | Teaching institutions  Hospital management  Nurse leaders (supervision and follow up)  Educators and preceptors (Good role models for juniors and students) | - Behavior changes among all concerned personnel  - Clinical mastering of PPM  - pediatric patients free of pain (zero pain in hospitals) |
| Policy and guidelines on PPM:  No Policy on PPM  Pain management guidelines (2012); general and does not guide on PPM. | Develop, review, and disseminate PPM policy, guidelines, and protocols | Ministry of health/RBC  Hospitals | Set policy, guidelines, and protocols to refer for effective PPM |
| Lack of clinical decision autonomy among nurses:  At secondary and tertiary health facilities, nurse executes orders and/or seeks approval of physician for PPM interventions. Unclear scope of nursing practice toward responsibilities of nurse specialists/ or depending on their education level leading to lack of clinical decision autonomy among nurses | Empowering nurses:   - Involvement of nurses as a collaborative practice in the decision-making regarding pain relief practices - Update and refinement of the nursing scope of practice to specify nursing responsibility, qualification & clinical decision autonomy about PPM | NCNM/RNMU for the scope of practice refinement  Hospitals for implementation | Nurses become autonomous in deciding PPM within their scope of practice |
| Poor collaboration between nurse educators and preceptors:  Inconsistency of what is taught to students at teaching institutions by nurse educators and in clinical settings by Preceptors | Improving collaboration among nurse educators and preceptors  Nurse clinicians to teach nursing students to share lived experience; Nurse educators should have recognized and regular clinical practices to enhance clinical experiences  Nurse educators updated on policy, guidelines, and new protocols for PPM | Teaching institutions  Hospital management  Educators and preceptors | A well-established collaboration between nurse educators and nurse preceptors |

Abbreviations: PPM, pediatric pain management; NCNM, national council for nurses and midwives; RNMU; Rwanda nurses and midwives’ union; RBC, Rwanda biomedical Centre
